# Targeting pro-dopaminergic agonism to attenuate depression in patients displaying genetic/epigenetic predisposition to hypodopaminergia

**DOI:** 10.3389/fpubh.2025.1594872

**Published:** 2025-09-25

**Authors:** Kai-Uwe Lewandrowski, Kenneth Blum, Alexander P. L. Lewandrowski, Panayotis K. Thanos, Albert Pinhasov, Alireza Sharafshah, David Baron, Mark S. Gold, Catherine A. Dennen, Igor Elman, Aballa Bowirrat, Edward J. Modestino, Foojan Zeine, Nicole Jafari, Keerthy Sunder, Milan T. Makale, John Giordano, Marjorie C. Gondre-Lewis, Marco Lindenau, Brian S. Fuehrlein, Rajendra D. Badgaiyan, Chynna Levin, Sergio Luis Schmidt, Rossano Kepler Alvim Fiorelli

**Affiliations:** 1 Division of Personalized Genomics, The Blum Institute of Neurogenetics & Behavior, Austin, TX, United States; 2 Department of Orthopedic Surgery, University of Arizona Tucson Campus, Tucson, AZ, United States; 3 Department of Orthopaedics, Fundación Universitaria Sanitas, Bogotá, Colombia; 4 Department of Orthopedics, Hospital Universitário Gaffree Guinle Universidade Federal do Estado do Rio de Janeiro, Rio de Janeiro, Brazil; 5 Division of Personalized Pain Research and Education, Center for Advanced Spine Care of Southern Arizona, Tucson, AZ, United States; 6 Department of Molecular Biology and Adelson School of Medicine, Ariel University, Ariel, Israel; 7 Division of Addiction Research & Education, Center for Sports, Exercise, and Mental Health, Western University of Health Sciences, Pomona, CA, United States; 8 Brain & Behavior Lab, Department of Psychology, Curry College, Milton, MA, United States; 9 Division of Recovery and Rehabilitation, JC Recovery and Counseling Center, Hollywood, FL, United States; 10 Faculty of Education and Psychology, Institute of Psychology, Eötvös Loránd University, Budapest, Hungary; 11 Department of Psychiatry, University of Vermont, Burlington, VT, United States; 12 Division of Neuromodulation Research, Karma Doctors & Karma TMS, Palm Springs, CA, United States; 13 Department of Biological Sciences, Dornsife College of Letters, Arts & Sciences, University of Southern California, Los Angeles, CA, United States; 14 Behavioral Neuropharmacology and Neuroimaging Laboratory on Addictions, Department of Pharmacology and Toxicology, Jacobs School of Medicine and Biosciences, Clinical Research Institute on Addictions, State University of New York at Buffalo, Buffalo, NY, United States; 15 Cellular and Molecular Research Center, School of Medicine, Guilan University of Medical Sciences, Rasht, Iran; 16 Department of Psychiatry & Behavioral Sciences, Stanford University School of Medicine, Palo Alto, CA, United States; 17 Department of Psychiatry, Washington University School of Medicine, St. Louis, MO, United States; 18 Department of Family Medicine, Jefferson Health Northeast, Philadelphia, PA, United States; 19 Department of Psychiatry, Cambridge Alliance, Harvard University School of Medicine, Cambridge, MA, United States; 20 Department of Health Science, California State University at Long Beach, Long Beach, CA, United States; 21 Department of Applied Clinical Psychology, The Chicago School of Professional Psychology, Los Angeles, CA, United States; 22 Department of Psychiatry, University of California, Riverside, Riverside, CA, United States; 23 Department of Radiation Medicine and Applied Sciences, University of California, San Diego, La Jolla, CA, United States; 24 Department of Anatomy, Howard University College of Medicine, Washington, DC, United States; 25 Department of Psychiatry, Yale University, New Haven, CT, United States; 26 Department of Psychiatry, School of Medicine, Texas Tech University Health Sciences Center, Midland, TX, United States; 27 Department of Psychiatry, Mt. Sinai University School of Medicine, New York, NY, United States; 28 Department of Clinical Psychology, St. John's University, Queens, NY, United States

**Keywords:** dopamine D2 receptor (DRD2), hypodopaminergia, pro-dopaminergic therapy, stress induced anxiety, genetic predisposition, Reward Deficiency Syndrome (RDS), neurotransmitter regulation, addiction and mood disorders

## Introduction

Since 1990, substantial evidence from association studies has identified the D(2) dopamine receptor (DRD2) gene as a factor in the development of alcoholism (1–4). The DRD2 gene has also been linked to other substance use disorders, including dependencies on cocaine, nicotine, and opioids, as well as obesity (5–11). Dopamine in the brain, often referred to as the “stress-relief molecule,” plays a central role in managing stress responses (12).

The relationship between dopaminergic neurotransmission and various forms of stress has been known for many years. The current understanding is that numerous genes interacting with dopaminergic pathways may comprise promising therapeutic targets, particularly in addiction treatment (13). Li et al. identified 396 genes that together influence dopamine and glutamate release in addiction contexts (14). The consistent evidence supporting dopamine's role in addiction has driven the development of therapies focused on modulating dopaminergic signaling (7).

### Dopamine D2 receptor neuro-genetics and auto- receptor function

A significant limitation in suppressing the dopaminergic system to induce drug extinction is the potential for mood disturbances and an increased risk of suicidal ideation. These side effects are counter-productive to the aim of the approach. Our laboratory has proposed that long-term, gentle stimulation of dopamine receptors could induce the “normalization” of reduced dopamine D2 receptor density (15).

Our laboratory has promoted the extended–term use of dopaminergic agonist therapies to reduce cravings for substances such as glucose based on the understanding that individuals carrying the DRD2 Taq A1 allele exhibit compromised D2 receptor density (16, 17). Positron emission tomography (PET) imaging studies have revealed substantial variability in dopamine D2 receptor density across *in vivo* human striatum. Low D2 receptor binding *in vivo* has been consistently associated with dependence on alcohol and other substances. The DRD2 A1 allele has been potentially linked to a subtype of alcoholism and reduced D2 receptor density *in vitro*. Pohjalainen et al. (18) conducted a study involving 54 healthy Finnish participants using PET imaging with [11C] raclopride to evaluate D2 receptor characteristics, including binding density (Bmax), affinity (Kd), and availability (Bmax/Kd). They observed that the A1/A2 genotype group exhibited significantly reduced D2 receptor availability compared to the A2/A2 group, indicating an alteration in receptor density. No difference in receptor affinity (Kd) was observed between the groups. The association between the A1 allele and low D2 receptor availability in healthy subjects indicates that the A1 allele of the TaqIA polymorphism may be in linkage disequilibrium with a promoter/regulatory mutation affecting dopamine D2 receptor expression. This research provides an *in vivo* neurobiological correlation between the A1 allele and lower D2 receptor availability in healthy individuals, aligning with our laboratory's work to underscore the importance of targeted interventions to address the neurobiological underpinnings of dopamine dysfunction in individuals with genetic predispositions (17).

### Therapeutic implications of D2 receptor regulation

Understanding why D2 receptor density was lower in A1 allele carriers provided the impetus to suggest that raising D2 receptor density may reduce aberrant craving behavior, providing a homeostatic state toward normalization. This concept was initially supported by Boundy et al. (19), whose research with radiolabeled antagonists demonstrated that both agonists and antagonists could induce up-regulation of D2 dopamine receptors in cells transfected to express D2L or D2S receptors. Notably, receptor regulation induced by agonists was synergistic with cAMP analogs, and the time courses of the effects varied between agonists and antagonists. Further studies extended these findings by utilizing radiolabeled agonists to examine agonist- and antagonist-induced regulation of the high-affinity state of the D2L dopamine receptor in transfected HEK 293 cells. Exposure to agonists resulted in a reduction of receptors in the high-affinity agonist-preferring state, whereas antagonists increased the density of such receptors. The effects of both agonists and antagonists on the agonist-preferring receptors occurred without a lag and were time and dose-dependent. Forskolin-stimulated cAMP accumulation was unaffected by exposing cells to the antagonist (-)-sulpiride, revealing that antagonists do not inhibit cAMP activity. However, after 1.5 h of exposure to the agonist quinpirole, desensitization occurred. This suggests that the rapid loss of high-affinity binding sites represents an uncoupling of the receptor from the G protein that mediates the inhibition of adenylyl cyclase. Pretreatment of cells with the protein synthesis inhibitor cycloheximide did not prevent this quinpirole-induced loss of receptors with a high affinity for agonists. Cycloheximide blocked the (-)-sulpiride-induced increase in high-affinity binding sites, but only after extended incubation sufficient to upregulate total receptor numbers. Short-term incubation of cells with (-)-sulpiride in cycloheximide still presented an increased receptor density with high agonist affinity. These results suggest that the increase in agonist binding after brief exposure to an antagonist is due to interactions of the receptor with one or more G proteins that are not coupled to inhibition of adenylyl cyclase, whereas the increase in agonist binding at later time points is associated with the antagonist-induced up-regulation.

**Figure 1:**
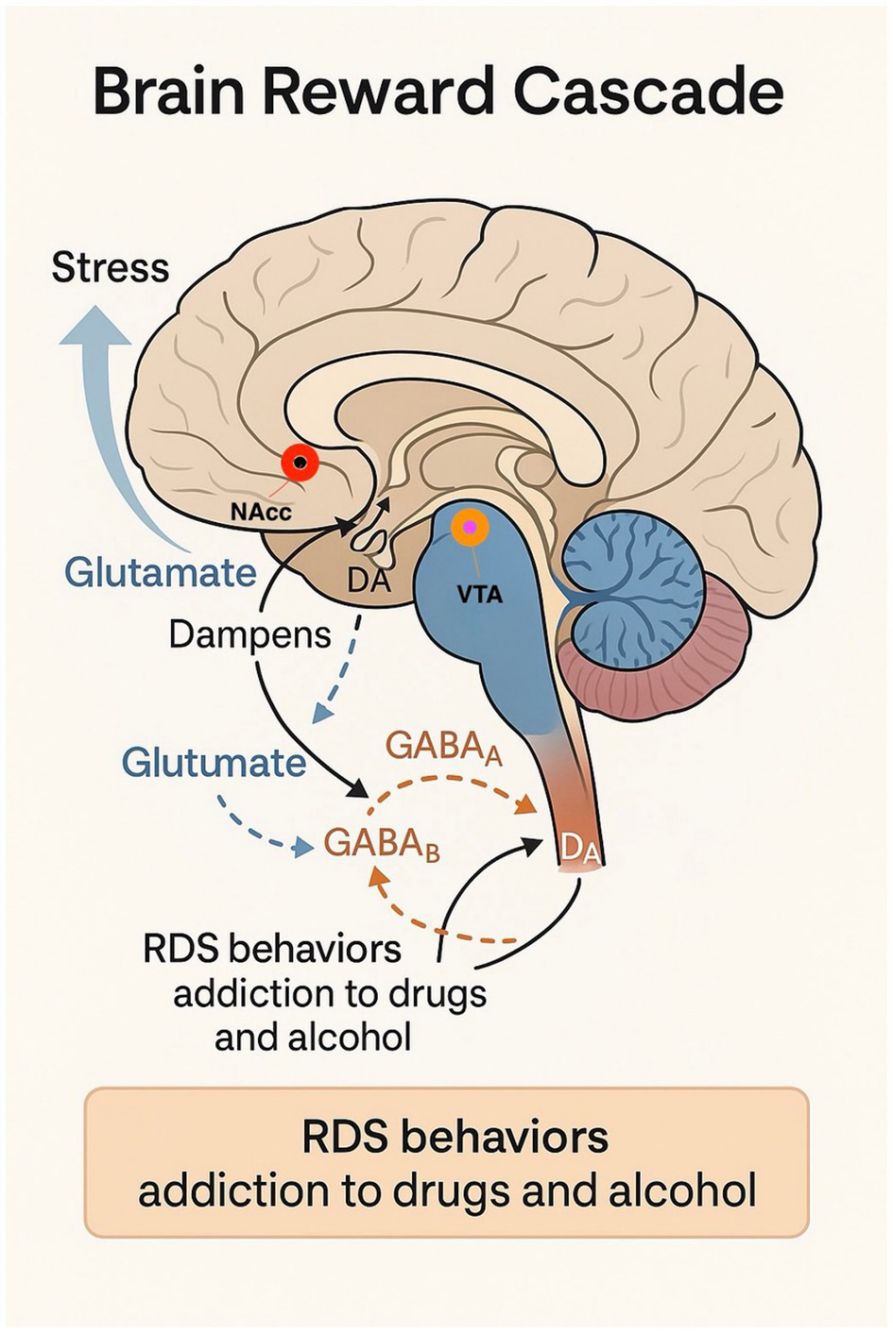
Brain Reward Cascade and the Neurobiological Basis of Reward Deficiency Syndrome (RDS). This schematic of the relevant pathways and neurotransmitter systems is simplified for clarity and illustrates the neurocircuitry of the Brain Reward Cascade (BRC), emphasizing the interactions between key neurotransmitter systems involved in stress modulation and dopaminergic signaling. Stress activates glutamatergic neurons in the prefrontal cortex (PFC), which project to the nucleus accumbens (NAcc) and ventral tegmental area (VTA). Increased glutamate (GLUT) release modulates dopamine (DA) release in the NAcc through NMDA receptor activation and downstream GABAergic regulation of VTA dopaminergic neurons via GABAA and GABAB receptors. Disruptions in this cascade—such as low D2 receptor density due to the DRD2 A1 allele—contribute to hypodopaminergia, a hallmark of Reward Deficiency Syndrome (RDS). RDS behaviors include substance and non-substance addictions (e.g., alcohol, drugs, gambling, overeating, internet use, and risk-taking behavior). This model supports the clinical rationale for genetic testing and precision-targeted dopaminergic modulation in managing addiction and stress-related disorders.

Thus, the gradual administration of agonistic therapy promotes the proliferation of Dopamine D2 receptors over time (20). This finding holds significant therapeutic potential, particularly in the use of KB220Z, a dopaminergic agonist reported to address Reward Deficiency Syndrome (RDS) behaviors (Figure 1), including addiction to substances such as drugs and alcohol (21). Studies indicate that individuals carrying the DRD2 A1 allele exhibit a higher likelihood of positive treatment response and compliance with dopaminergic agonist therapy compared to those with the DRD2 A2 allele genotype. However, it must be noted that the precise mechanisms producing these favorable clinical responses remain unclear (22–25).

Laakso et al. (26) provided critical insights into the mechanisms underlying dopamine dysfunction for the first time in the study of dopaminergic genetics. Their research indicates that the A1 allele of the *TaqI* restriction fragment length polymorphism (RFLP) in the dopamine D2 receptor gene (DRD2) is associated with reduced D2 receptor density in the striatum. Recognizing the key role of D2 autoreceptors in dopamine synthesis regulation, they investigated whether the A1 allele alters presynaptic dopamine function in the brain. They additionally studied two other DRD2 polymorphisms, C957T and−141C Ins/Del, which have also been suggested to affect D2 receptor levels in the brain. The relationships between the *Taq IA* RFLP, C957 T, and−141C Ins/Del polymorphisms and striatal dopamine synthesis in 33 healthy Finnish volunteers were studied using positron emission tomography and [18F] fluorodopa [[18F] FDOPA], a radiolabeled analog of the dopamine precursor L-DOPA. The study revealed that heterozygous carriers of the A1 allele (A1/A2; 10 subjects) exhibited an 18% increase in [18F] FDOPA uptake in the putamen compared to non-carriers (A2/A2; 23 subjects). In contrast, the C957T and−141C Ins/Del polymorphisms did not significantly affect [18F] FDOPA uptake values. These findings demonstrate that the A1 allele of the DRD2 gene is linked to the increased striatal activity of aromatic L-amino acid decarboxylase, the final enzyme in dopamine biosynthesis and the rate-limiting enzyme for trace amine (e.g., beta-phenylethylamine) synthesis (26). The increased activity of this enzyme is thought to compensate for lower D2 receptor expression caused by the A1 allele, leading to decreased autoreceptor function. These results suggest that dopamine synthesis in A1 allele carriers could benefit from a gentler, less potent dopaminergic agonist compared to L-DOPA. This supports the use of the KB220z complex, precursor amino acid, and enkephalinase therapy as an effective dopamine agonist. It is proposed that lower DA quanta dopamine release at presynaptic neurons in the N. accumbens should induce receptor upregulation in A1 allele carriers, ultimately reducing craving behaviors and contributing to dopamine homeostasis.

### Silent mutations and functional impact on DRD2 expression

In the article “The Price of Silent Mutations,” published in ^*^Scientific American^*^, Chamary and Hurst (27) posit that minor DNA changes previously thought innocuous may have profound implications for human diseases, evolution, and biotechnology. The article mentions silent mutations within the DNA code, revealing that mutations located outside gene regulatory introns can significantly influence how genes are translated into proteins. Over time, studies have linked the 3′ untranslated region (UTR) to mRNA activity, demonstrating its critical role in gene expression. Chamary and Hurst specifically identify a silent mutation in the dopamine D2 receptor (DRD2) gene, which encodes a receptor that detects the neurotransmitter dopamine. One silent mutation in this gene causes accelerated degradation of mRNA, resulting in reduced production of the encoded protein, which may, in turn, affect certain disease states.

This suggests that the DRD2 *Taq* A1 allele association in the 3′ region by Grandy and our subsequent association studies are due to synonymous mutations (silent) in the human dopamine D2 affect mRNA stability and thus synthesis of the receptor. Notably, mutations like−957T are now recognized as being connected to the Taq A1 allele (28). These findings challenge traditional assumptions concerning synonymous variations in molecular genetics and gene-mapping studies. In the context of complex inherited conditions, such as stress and RDS, synonymous variation may hold significant pathophysiological and pharmacogenetic relevance. This underscores the need for further research regarding silent mutations in genetic regulation and their broader implications.

### Neurobiological mechanisms of stress and dopamine dysregulation

A recent PUBMED search identified 13,003 articles related to dopamine (DA) (retrieved 11-18-24). Stress will stimulate dopamine (DA) transmission in both the medial prefrontal cortex (PFC) and the nucleus accumbens (NAcc) (29). However, the NAcc dopamine response to stress appears to be modulated by a DA-sensitive mechanism in the PFC, where increased DA transmission in this cortical region dampens the NAcc response to various stress stimuli (30). There is also evidence implicating PFC glutamate (GLUT)-producing neurons, some of which project to the NAcc and the ventral tegmental area (VTA), the origin of the mesocorticolimbic dopamine system (31, 32).

**Figure 2:**
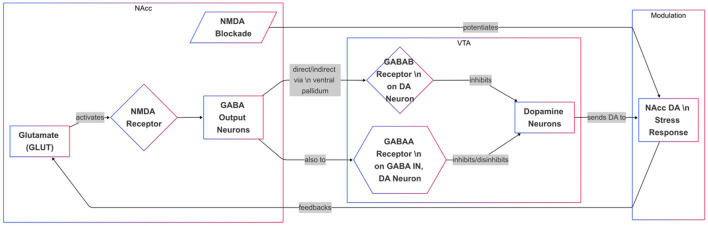
Regulation of the NAcc Dopamine Stress Response via Glutamate-GABA-VTA Circuitry. This diagram depicts how glutamate (GLUT) and GABA signaling influence dopamine (DA) release in the nucleus accumbens (NAcc) during stress. GLUT activates NMDA receptors in the NAcc, stimulating GABA output neurons that project directly or indirectly to the ventral tegmental area (VTA). In the VTA, GABAB receptors on DA neurons inhibit activity, while GABAA receptors on interneurons or DA neurons modulate DA firing through both inhibition and disinhibition. NMDA receptor blockade in the NAcc potentiates the DA stress response, suggesting a feedback loop involving GLUT, GABA, and VTA DA neurons. This circuitry ultimately regulates DA transmission in response to stress.

Stress not only enhances dopamine transmission but also elevates GLUT levels in the PFC and NAcc (33). Research indicates that the NAcc dopamine stress response is influenced by a GLUT-sensitive mechanism (34, 35). Furthermore, studies have shown that blocking NMDA receptors locally in the NAcc potentiates the dopamine stress response (36). This suggests that NMDA receptors on NAcc output neurons, which project to the VTA, mediate the local effects of GLUT on the NAcc DA stress response. Part of the NAcc output system comprises GABA neurons that project either directly or indirectly to the VTA via the ventral pallidum (37). In the VTA, GABA is known to hyperpolarize DA cells, inhibiting their activity through GABA_B_ receptor-mediated action. GABA also regulates VTA dopamine cells at GABA_A_ receptors, which exert both inhibitory and disinhibitory effects alongside predominant indirect disinhibitory action, likely via presynaptic action on non-dopaminergic interneurons (37). Local activation of GABA_A_ and GABA_B_ receptors in the VTA modulates dopamine transmission in both the NAcc and VTA (Figure 2). However, to our knowledge, no comparable studies have directly explored how these mechanisms affect the NAcc dopamine response, specifically under stress (37).

Evidence suggests that the dopamine (DA) stress response in the nucleus accumbens (NAcc) is regulated by GABA inputs to VTA dopamine, with differential effects mediated by GABA_A_ and GABA_B_ receptors (38). Data indicates that GABA_B_ receptors are located directly on DA neurons, while GABA_A_ receptors are found on GABA interneurons and potentially on DA neurons themselves. These findings align with the presumption that corticofugal glutamate (GLUT) inputs to the NAcc regulate stress-induced DA release indirectly through a GABA-mediated feedback pathway to the VTA.

### Genetic vulnerability, hypodopaminergia, and stress-induced addiction risk

Over the past decade, it has become increasingly clear that susceptibility to substance use disorders is influenced by complex interactions between genetic and environmental determinants (39–42). Notably, impulsive behaviors are more likely to occur under conditions of stress or heightened arousal (43). Well-supported associations between stress and substance abuse have been noted (44, 45). However, the precise nature of stress-induced alterations on DA neurotransmission, the conditions under which these alterations occur, and the ability to generalize the preclinical findings to humans remain to be determined.

Since Blum et al. (46) linked dopamine D2 receptor (DRD2) gene polymorphisms to severe alcoholism, subsequent research has associated DRD2 gene polymorphisms with both acute and chronic forms of stress. Importantly, emerging evidence underscores the role of genetic and epigenetic factors in creating a state of “hypodopaminergia,” which may increase susceptibility to trauma, as in post-traumatic stress disorder (PTSD) (47). A series of studies by the RDS Consortium provided evidence for DNA antecedents involving hypodopaminergia, highlighting its importance in RDS vulnerability and urging the scientific community to investigate the potential of induction of “dopamine” “homeostasis” with pro-dopamine regulation (e.g., KB220) (48–68).

**Figure 3:**
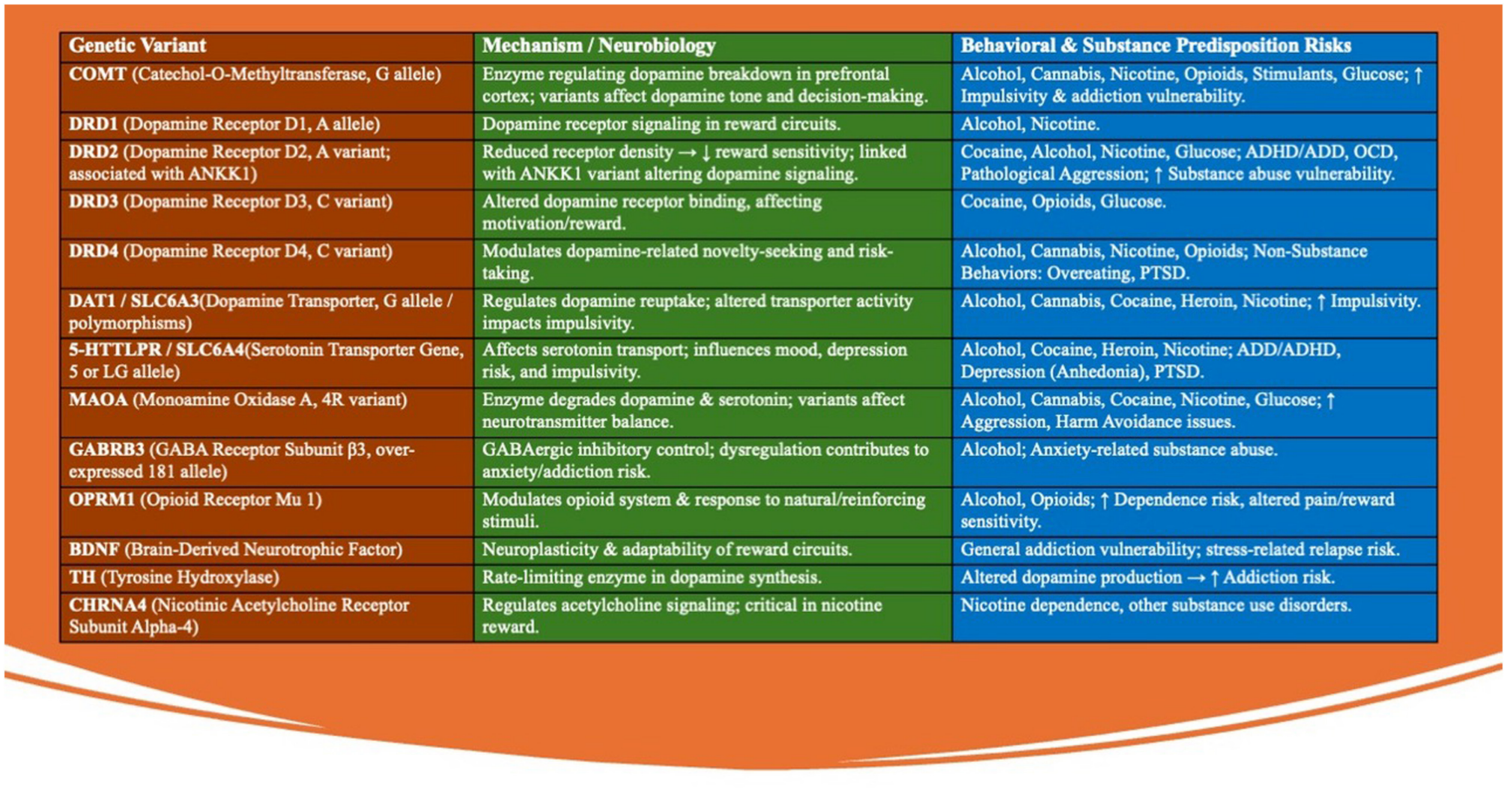
The Genetic Addiction Risk Score (GARS) test evaluates 10-12 specific genetic variants that are closely associated with the brain's reward pathways and the risk of addictive behaviors. These genetic variants collectively contribute to an individual's predisposition to addictive behaviors by affecting the brain's reward circuitry. The GARS test assesses the presence and combination of these variants to provide a genetic risk score that can be used to guide personalized interventions and prevention strategies. These genetic variants are found in key neurotransmitter systems, including dopamine, serotonin, and endorphins, which play crucial roles in regulating mood, motivation, and pleasure. The following are some of the key genetic variants tested in the GARS assessment: (1) DRD2 (Dopamine Receptor D2): Variants in this gene are linked to reduced dopamine receptor density, leading to decreased reward sensitivity and an increased risk of substance abuse and other addictive behaviors. (2) DAT1 (Dopamine Transporter): This gene regulates dopamine reuptake in the brain. Certain polymorphisms can result in altered dopamine availability, contributing to impulsivity and the propensity for addiction. (3) ANKK1 (Ankyrin Repeat and Kinase Domain Containing 1): Often associated with the DRD2 gene, variations in ANKK1 influence dopamine receptor signaling and have been linked to higher risks of addiction and compulsive behaviors. (4) COMT (Catechol-O-Methyltransferase): This enzyme is involved in the breakdown of dopamine. Variants in the COMT gene can affect dopamine levels in the prefrontal cortex, impacting decision-making and increasing susceptibility to addictive behaviors. (5) MAOA (Monoamine Oxidase A): This gene encodes an enzyme that breaks down neurotransmitters like dopamine and serotonin. Certain variants can lead to imbalances in these neurotransmitters, contributing to impulsivity and addiction risk. (6) OPRM1 (Opioid Receptor Mu 1): Variants in this gene affect the opioid system, influencing pain perception and the rewarding effects of substances like alcohol and opioids, thereby increasing the likelihood of addiction. (7) BDNF (Brain-Derived Neurotrophic Factor): This gene is involved in neuroplasticity. Variants in BDNF can affect the brain's ability to adapt to new experiences, potentially increasing vulnerability to addictive behaviors. (8) 5HTTLPR (Serotonin Transporter Gene): Polymorphisms in this gene affect serotonin transport and are linked to mood disorders and increased risk-taking behaviors, which can contribute to addiction. (9) GABRB3 (Gamma-Aminobutyric Acid Receptor Subunit Beta-3): Variants in this gene influence the GABAergic system, which is critical for inhibitory signaling in the brain. Dysregulation here can lead to anxiety and susceptibility to substance abuse. (10) TH (Tyrosine Hydroxylase): This gene is involved in the synthesis of dopamine. Variants in TH can influence dopamine production, affecting reward processing and increasing addiction risk. (11) SLC6A3 (Solute Carrier Family 6 Member 3): This gene encodes the dopamine transporter protein, and its variants can affect dopamine reuptake, contributing to altered dopamine signaling and an increased risk of addictive behaviors. (12) CHRNA4 (Cholinergic Receptor Nicotinic Alpha 4 Subunit): Variants in this gene are associated with nicotine dependence and other substance use disorders due to its role in acetylcholine receptor function in the brain.

This growing body of evidence underscores the need for targeted interventions to address the interplay between stress, dopamine regulation, and genetic predisposition, paving the way for precision therapies aimed at restoring dopamine balance in affected individuals. The Genetic Addiction Risk Score (GARS) test allows to quantify the risk of addictive behaviors (Figure 3).

## Conclusion

Stress is widely recognized as a significant risk factor for the onset of addiction, chronic pain, and vulnerability to relapse. Population-based and epidemiological studies have identified specific stressors and individual-level variables that are predictive of substance use and abuse. Preclinical studies further demonstrate that stress exposure increases drug self-administration and reinstates drug-seeking behavior in previously drug-experienced animals. The deleterious impact of early life stress, child maltreatment, and accumulated adversity on the corticotropin-releasing factor/hypothalamic-pituitary-adrenal axis (CRF/HPA), extrahypothalamic CRF, autonomic arousal, and central noradrenergic systems are reported to be relevant.

Noradrenergic activation is closely tied to the severity of stress experienced. The effects of these alterations on the corticostriatal-limbic motivational, learning, and adaptation systems that include mesolimbic dopamine, glutamate, and gamma-amino-butyric acid (GABA) pathways are all associated with the underlying pathophysiology linked with stress-related risk of addiction.

Although significant research gaps remain in understanding the precise relationship between stress and addiction, existing literature highlights a promising non-pharmacological approach-KB220. This pro-dopaminergic compound has the potential to drive new prevention and treatment plans to address stress-induced vulnerability associated with hypodopaminergia. The novel approach may mitigate reward deficiency and reduce the likelihood of substance- and non-substance-related addictive behaviors.
