# Correction: Targeting pro-dopaminergic agonism to attenuate depression in patients displaying genetic/epigenetic predisposition to hypodopaminergia

**DOI:** 10.3389/fpubh.2025.1737084

**Published:** 2025-11-17

**Authors:** Kai-Uwe Lewandrowski, Kenneth Blum, Alexander P. L. Lewandrowski, Panayotis K. Thanos, Albert Pinhasov, Alireza Sharafshah, David Baron, Mark S. Gold, Catherine A. Dennen, Igor Elman, Aballa Bowirrat, Edward J. Modestino, Foojan Zeine, Nicole Jafari, Keerthy Sunder, Milan T. Makale, John Giordano, Marjorie C. Gondre-Lewis, Marco Lindenau, Brian S. Fuehrlein, Rajendra D. Badgaiyan, Chynna Levin, Sergio Luis Schmidt, Rossano Kepler Alvim Fiorelli

**Affiliations:** 1 Division of Personalized Genomics, The Blum Institute of Neurogenetics & Behavior, Austin, TX, United States; 2 Department of Orthopedic Surgery, University of Arizona Tucson Campus, Tucson, AZ, United States; 3 Department of Orthopaedics, Fundación Universitaria Sanitas, Bogotá, Colombia; 4 Department of Orthopedics, Hospital Universitário Gaffree Guinle Universidade Federal do Estado do Rio de Janeiro, Rio de Janeiro, Brazil; 5 Division of Personalized Pain Research and Education, Center for Advanced Spine Care of Southern Arizona, Tucson, AZ, United States; 6 Department of Molecular Biology and Adelson School of Medicine, Ariel University, Ariel, Israel; 7 Division of Addiction Research & Education, Center for Sports, Exercise, and Mental Health, Western University of Health Sciences, Pomona, CA, United States; 8 Brain & Behavior Lab, Department of Psychology, Curry College, Milton, MA, United States; 9 Division of Recovery and Rehabilitation, JC Recovery and Counseling Center, Hollywood, FL, United States; 10 Faculty of Education and Psychology, Institute of Psychology, Eötvös Loránd University, Budapest, Hungary; 11 Department of Psychiatry, University of Vermont, Burlington, VT, United States; 12 Division of Neuromodulation Research, Karma Doctors & Karma TMS, Palm Springs, CA, United States; 13 Department of Biological Sciences, Dornsife College of Letters, Arts & Sciences, University of Southern California, Los Angeles, CA, United States; 14 Behavioral Neuropharmacology and Neuroimaging Laboratory on Addictions, Department of Pharmacology and Toxicology, Jacobs School of Medicine and Biosciences, Clinical Research Institute on Addictions, State University of New York at Buffalo, Buffalo, NY, United States; 15 Cellular and Molecular Research Center, School of Medicine, Guilan University of Medical Sciences, Rasht, Iran; 16 Department of Psychiatry & Behavioral Sciences, Stanford University School of Medicine, Palo Alto, CA, United States; 17 Department of Psychiatry, Washington University School of Medicine, St. Louis, MO, United States; 18 Department of Family Medicine, Jefferson Health Northeast, Philadelphia, PA, United States; 19 Department of Psychiatry, Cambridge Alliance, Harvard University School of Medicine, Cambridge, MA, United States; 20 Department of Health Science, California State University at Long Beach, Long Beach, CA, United States; 21 Department of Applied Clinical Psychology, The Chicago School of Professional Psychology, Los Angeles, CA, United States; 22 Department of Psychiatry, University of California, Riverside, Riverside, CA, United States; 23 Department of Radiation Medicine and Applied Sciences, University of California, San Diego, La Jolla, CA, United States; 24 Department of Anatomy, Howard University College of Medicine, Washington, DC, United States; 25 Department of Psychiatry, Yale University, New Haven, CT, United States; 26 Department of Psychiatry, School of Medicine, Texas Tech University Health Sciences Center, Midland, TX, United States; 27 Department of Psychiatry, Mt. Sinai University School of Medicine, New York, NY, United States; 28 Department of Clinical Psychology, St. John's University, Queens, NY, United States

**Keywords:** dopamine D2 receptor (DRD2), hypodopaminergia, pro-dopaminergic therapy, stress induced anxiety, genetic predisposition, Reward Deficiency Syndrome (RDS), neurotransmitter regulation, addiction and mood disorders

The title of this article was erroneously given as “Targeting pro-dopaminergic agonism to attenuate and depression in patient's displaying genetic/epigenetic predisposition to hypodopaminergia.”

The correct title of the article is “Targeting pro-dopaminergic agonism to attenuate depression in patients displaying genetic/epigenetic predisposition to hypodopaminergia.”

The original version of this article has been updated.

